# Evidence for an interaction of paracingulin with microtubules

**DOI:** 10.17912/micropub.biology.001341

**Published:** 2024-10-14

**Authors:** Arielle Flinois, Annick Mutero-Maeda, Sylvie Montessuit, Sandra Citi

**Affiliations:** 1 University of Geneva, Geneva, Switzerland

## Abstract

The mechanisms that anchor microtubules to epithelial junctions are poorly understood. Here we show that recombinant purified paracingulin ( CGNL1 , JACOP), a cytoplasmic junctional protein, decorates microtubules by negative staining electron microscopy and co-pellets with microtubules. Co-pelleting experiments using fragments of CGNL1 indicate that this is mediated by a central region of the CGNL1 head domain (residues 250-420). Deletion of a basic amino-acid stretch (365-377) within this fragment, abolishes both co-pelleting with and decoration of microtubules. These results suggest that paracingulin can interact directly with microtubules through a basic amino-acid stretch of its head domain.

**Figure 1. CGNL1 decorates and co-pellets with microtubules through a basic-rich region in the head domain:**
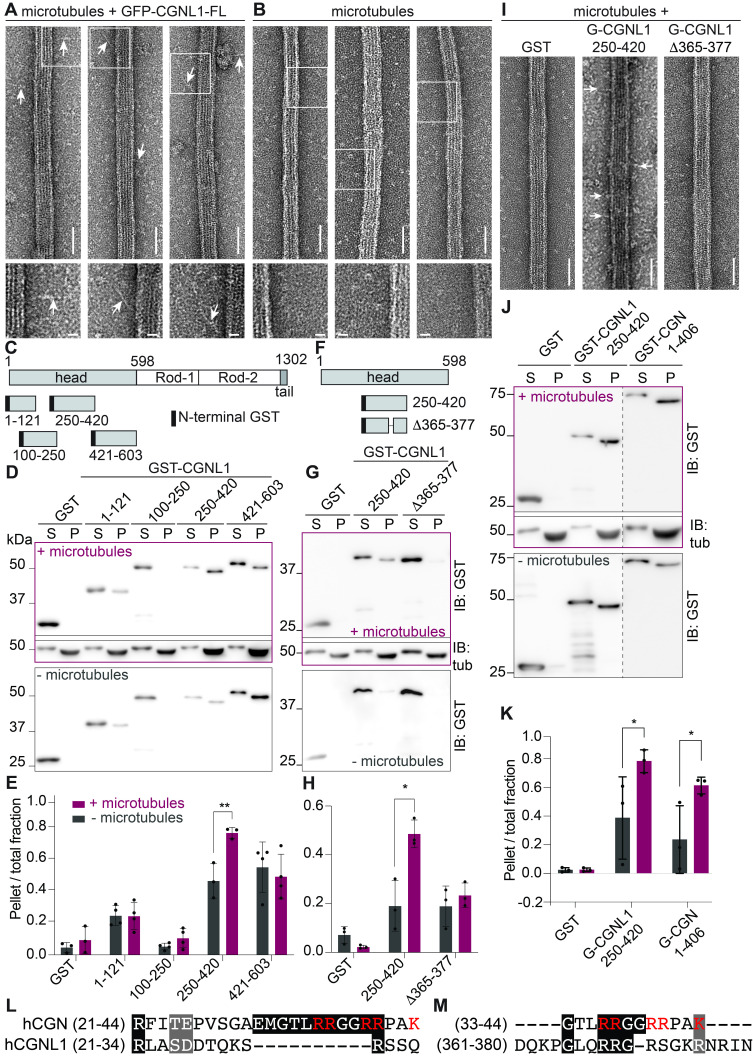
(A-B) Electron microscopy analysis of negatively stained samples of microtubules incubated with GFP-tagged CGNL1 (A) or microtubules alone (B). Insets at the bottom show high magnification details of CGNL1 rod projections from the microtubule shaft, outlined by square boxes on low magnification images. Arrows indicate elongated rod domains on the side of microtubules. Scale bars are 50 nm in low magnification and 10 nm in insets. (C) Simplified scheme of structural domains (head, rod, tail) of human CGNL1, and GST fusion constructs of the head domain used for MT-pelleting assay, indicating amino acid residue boundaries. (D) IB analysis, using anti-GST and anti-tubulin antibodies, of a representative MT-pelleting assay using GST, or the GST-fused constructs described in (C) in the presence (purple) or the absence of MTs (grey). For each sample, supernatant (S) and pellet (P) fractions are loaded next to each other to compare the relative amount of protein in each fraction. (E) Quantification of the amount of protein in the pellet fraction, ratioed to the total amount of protein in both the pellet and supernatant fractions (N = 3-4 biological replicates). (F) Simplified scheme of structural domains (head, rod, tail) of human CGNL1, and GST fusion construct of the (250-420) fragment, either WT or with an internal deletion (Δ365-377) corresponding to the sequence highlighted in panel M (bottom). (G) IB analysis, using anti-GST and anti-tubulin antibodies, of a representative MT-pelleting assay using GST, or the GST-fused constructs described in (G) in the presence (purple, top) or the absence of MTs (grey, bottom). For each sample, supernatant (S) and pellet (P) fractions are loaded next to each other to compare the relative amount of protein in each fraction. (H) Quantification of the amount of protein in the pellet fraction, ratioed to the total amount of protein in both the pellet and supernatant fractions (N = 3 biological replicates). (I) Electron microscopy analysis of negatively stained samples of microtubules incubated with either GST, GST-tagged CGNL1(250-420) fragment, either WT or with an internal deletion (Δ365-377). White arrows indicate microtubule decoration. Scale bars = 50 nm. (J) IB analysis, using anti-GST and anti-tubulin antibodies, of a representative MT-pelleting assay using either GST, or the GST-tagged MT-interacting fragment of CGNL1 (250-420) or the head region of cingulin (CGN (1-406)) (G) in the presence (purple, top) or the absence (grey, bottom) of MTs. For each sample, supernatant (S) and pellet (P) fractions are loaded next to each other to compare the relative amount of protein in each fraction. (K) Quantification of the amount of protein in the pellet fraction, ratioed to the total amount of protein in both the pellet and supernatant fractions (N = 3 biological replicates). For all quantifications of immunoblot signals (E, H, K), two-way ANOVA with post hoc Sidak's test (*p < 0.05) was used, and asterisks indicate statistical significance. (L-M) Alignments between the N-terminal residues of CGN and CGNL1 (L), and the region (361-419) of CGNL1 with the MT-interacting region of CGN (M) (Mangan et al, 2016). Sequence alignments were performed using the multiple sequence alignment of Clustal Omega (EMBL-EBI). Black and grey highlight indicates sequence identity and similarity, respectively, and basic residues within the cingulin MT-interacting region are shown in red.

## Description

In epithelial cells most microtubules (MTs) are non-centrosomal and are aligned along the apico-basal axis, with the minus ends oriented towards the apical pole. The rearrangement of the MT network during epithelial cell polarization is not fully understood, but the formation of cell-cell junctions appears to be a crucial pre-requisite (Musch 2004). Among several junctional proteins that have been reported to interact either directly or indirectly with MTs (Vasileva and Citi 2018) , the tight junction (TJ) protein cingulin ( CGN ) was reported to organize the Planar Apical Network (PAN) of MTs in Eph4 cells and promote apical lumen formation during cyst morphogenesis of MDCK cells by binding to MTs (Yano et al. 2013; Mangan et al. 2016) . The CGN paralog paracingulin ( CGNL1 ) is localized at TJ and adherens junctions (AJ) and it recruits of a population of MTs to TJs by binding to the minus-end MT-binding protein CAMSAP3 (Flinois et al. 2024) . Although purified full-length CGNL1 co-pellets with microtubules in a pelleting assay (Vasileva and Citi 2018) , the CGN L1-MT interaction has not yet been confirmed by alternative methods and it is not known what sequences of CGN L1 are involved in its putative interaction with MTs.

To address these questions, we prepared taxol-stabilized MTs and incubated them with full-length recombinant GFP-tagged CGNL1 , purified from insect cells (Rouaud et al. 2023) , for analysis of layered MTs by negative staining electron microscopy. The elongated rod domains of CGNL1 were clearly detectable as projections emanating from the MT shafts (arrowheads and magnified insets, Figure 1A ), suggesting that CGNL1 can decorate the MT lattice, although not in a homogeneous and dense manner. In contrast, MTs alone did not harbor any projections along their lattice ( Figure 1B ). These results provide electron microscopy evidence for an interaction between MTs and CGNL1 .

To determine the region of CGNL1 involved in interaction with MTs, we considered previous evidence that in the case of CGN the MT-binding region is within the globular head domain (Yano et al. 2013; Mangan et al. 2016) . Given the sequence homologies and similar structure/function relationships between CGN and CGNL1 (Ohnishi et al. 2004; Guillemot and Citi 2006; Rouaud et al. 2020) and the detection of the rod domains projecting from the MT lattice ( Fig. 1A ), we hypothesized that the globular head domain of CGNL1 is involved in the interaction with MTs. Thus, we examined how bacterially expressed, purified GST-tagged fragments of the head domain of CGNL1 interact with MTs, using a co-pelleting assay ( Figure 1C ). Immunoblot analysis indicated that one fragment (250-420) was significantly enriched in the pellet fraction in the presence of MTs ( Figure 1D ). Quantification of the amount of protein in the pellet versus total fractions showed a significant increase of the (250-420) fragment in the presence versus absence of MTs ( Figure 1E ). For comparison, we carried out co-pelleting experiments with the head domain of CGN . Immunoblot analysis showed a similar fraction of protein in the MT-associated pellet when comparing the head of CGN (1-406) to the CGNL1 (250-420) fragment ( Figure 1J -K), suggesting similar binding affinities. Together, these results suggest that the interaction between CGNL1 and MTs requires the central region of the head domain of CGNL1 .

To identify more precisely the sequence of CGNL1 involved in its association with MTs, we aligned the head domains of CGN and CGNL1 . The MT-interacting region of CGN is a basic amino-acid rich stretch in the N-terminus (Mangan et al. 2016) . No sequence homology was detected between this sequence and the corresponding N-terminal sequence of CGNL1 ( Figure 1L ). However, alignment of the CGN MT-binding region with the whole head sequence of CGNL1 revealed a homology between residues 1-12 of CGN and residues 365-377 of head domain of CGNL1 ( Figure 1M ). Since this stretch lies within the (250-420) fragment that we identified by the co-pelleting assay ( Fig. 1C -E), we tested the relevance of this sequence by generating a mutated GST-tagged fragment, harboring a deletion of this sequence (Δ365-377; Figure 1F ). Next, we compared WT and mutated GST fragments for their ability to co-pellet with MTs, by immunoblot analysis ( Figure 1G ) and quantification of the ratio between pellet fraction and total fraction ( Figure 1H ). The results showed that the deletion mutant (Δ365-377) of the MT-interacting fragment (250-420) failed to co-pellet with MTs, in contrast to the WT (250-420) fragment ( Fig. 1G -H). In agreement with this observation, only the WT fragment, but not the deletion mutant or GST alone, was able to decorate MTs, as determined by negative staining electron microscopy ( Figure 1I ). Together, these results suggest that a basic amino-acid stretch within the central part of the head domain of CGNL1 is required for CGNL1 interaction with MTs in vitro.

It is noteworthy that CGNL1 did not decorate MTs completely and homogeneously, suggesting that the affinity of interaction is weak, compared to other characterized microtubule-associated proteins (MAPs) that decorate MTs more homogeneously (Ackmann, Wiech, and Mandelkow 2000; Miranda, King, and Harrison 2007) . This suggests that optimal CGN L1-MTs interaction may require either specific post-translational modification of CGN L1 or, such as phosphorylation, or of MTs, or additional molecular components. For example, phosphorylation of CGN by AMPK was shown to modulate its binding to either actin or microtubule filaments (Yano et al. 2018) . In addition, Microtubule Affinity Regulating Kinases (MARKs) can phosphorylate MAPs, such as tau and MAP4 , to regulate microtubule dynamics (Drewes et al. 1997) . Interestingly, specific MARK isoforms are localized at junctions and along lateral membranes of polarized epithelial cells and are required for establishment of apico-basal polarity (Suzuki et al. 2004; Cohen et al. 2011) , suggesting a potential interaction and reciprocal regulation between MARK proteins and junctional proteins such as CGN and CGN L1.

The physiological relevance of the interaction between MTs and either CGN or CGNL1 in epithelial cells and in tissue morphogenesis should be investigated by additional in vitro and in vivo studies. In the case of CGN , studies in vitro suggest that CGN regulates MT organization and epithelial morphogenesis by directly binding to MTs (Yano et al. 2013; Mangan et al. 2016) . However, there is so far no evidence for a role of CGN in regulating MT organization in vivo. In the case of CGNL1 , we provided evidence that CGNL1 depletion affects MT organization and epithelial polarity both in vitro and in vivo, in mouse tissues (Flinois et al. 2024) , suggesting non-redundant functions of CGNL1 and CGN , at least some cell types. Moreover, although studies in vitro indicate that junctional recruitment of the MT minus-end binding protein CAMSAP3 can account for some of the phenotypes of CGN L1-KO cells, the disruption of the PAN due to CGN L1-KO was independent of the region of CGN L1 that interacts with CAMSAP3 (Flinois et al. 2024) . Thus, the present study, by providing evidence for a potential direct in vitro interaction of CGNL1 with MTs, raises the hypothesis that CGNL1 may also function by binding directly to MTs in cells. Further studies are required to test this hypothesis and investigate how CGN and CGNL1 regulate epithelial morphogenesis through interaction with both the MT and actomyosin cytoskeleton and their regulators, such as GEFs, GAPs and MARKs (Rouaud et al. 2020; Rouaud et al. 2023; Citi et al. 2024) .

## Methods

**Antibodies**

The primary antibodies targeting the following proteins were used at the indicated dilution for immunoblotting (IB): rabbit GST tag (71-7500; Thermo Fisher Scientific; IB: 1/2000); mouse β-tubulin (32-2600; Thermo Fisher Scientific; IB: 1/3500) (see also Reagents).

**Plasmids**

GST-tagged fragments of CGNL1 and CGN in pGEX4T1 were described previously (Guillemot et al. 2008; Mangan et al. 2016) (Table S1). In addition, GST-tagged CGNL1 (Δ365-377) was obtained by PCR on CGNL1 (250-420) and cloned into pGEX4T1 (BamHI-XhoI) (S3006). For insect cell expression, the CGNL1 construct was generated by PCR amplification and subcloned into the indicated cloning sites: 2xStrep-10xHis-TEV-EGFP-h CGNL1 (FL) (1-1302aa; S2906) KpnI in pACEBac1. All constructs were validated by sequencing (Microsynth, Switzerland).

**Protein expression and purification**

GST-tagged proteins were expressed in BL21 as described in (Sluysmans et al. 2021) . Bacterial pellets derived from 25 ml of bacterial culture were resuspended in 1 ml of PBS, 1X PIC, 0.1% Tx-100, sonicated and centrifuged for 15 min at 4°C and the supernatant was used for subsequent purification. Glutathione magnetic beads (Thermo Fisher Scientific; 78602) were activated by washing twice in equilibration buffer (Tris-HCl 125 mM, pH 7.4/NaCl 150 mM/DTT 1 mM/EDTA 1 mM), and incubated with the clarified cell lysate for 2 hours at RT under rotation. The beads were then washed twice with equilibration buffer and the protein was eluted by incubating the beads with elution buffer (25 mM PIPES pH 7.8, 0.3 mM NaCl, 1 mM EDTA, 1 mM DTT, 50 mM glutathione, 0.1% Tx-100) for 1h at RT under rotation. Eluates were supplemented with 5% glycerol, aliquoted, snap frozen in liquid nitrogen, and stored at -80°C.

Full-length GFP-tagged h CGNL1 was purified from insect cell lysates as described in (Rouaud et al. 2023) . SDS-PAGE analysis of insect cell lysate and purified protein is shown in extended data image.

**Microtubule-pelleting assay**

Porcine tubulin (Cytoskeleton Inc.; T240) was polymerized at a concentration of 5 mg/ml in General Tubulin Buffer (GTB: 25 mM PIPES pH 7, 1 mM MgCl _2_ , 1 mM EGTA) in the presence of 5% glycerol and 1 mM GTP (Cytoskeleton Inc.; BST06) for 30 min at 37°C. An increasing amount of paclitaxel (Focus Biomolecules; FBM-10-2095) was added after 10 min (20 nM), 15 min (200 nM), and 20 min (2 µM) of incubation at 37°C to facilitate polymerization. After polymerization, microtubules (MTs) were diluted in GTB + 20 µM paclitaxel to a final concentration of 0.5 mg/ml. MT co-pelleting assays were performed by mixing 20 µl of purified GST-tagged CGNL1 fragments (0.1 mg/ml) in 25 mM PIPES pH 7.8, 300 mM NaCl, 1 mM DTT, 1 mM EDTA, with 20 µl of MTs or 20 µl of GTB buffer + 20 µM paclitaxel and adjusted to 50 µl total volume with GTB + 20 µM paclitaxel. All samples were incubated for 30 min at RT and then spun over 100 µl of Cushion Buffer (CB: 25 mM PIPES pH 7, 2 mM MgCl _2_ , 1 mM EGTA, 60% glycerol, 20 µM paclitaxel) at 16,000 x g for 40 min at RT. The supernatant (50 µl) was recovered and mixed with 10 µl of sample buffer (SB) 5X, and the pellet resuspended in 50 µl of SB 1X. All samples were then boiled for 5 min at 95°C and stored at -20°C for analysis by SDS-PAGE. In microtubule pelleting assays ( Figure 1D, G and J) (Hyman et al, 1991) , an immunoblot of tubulin is shown for the conditions where the protein is mixed with microtubules to ensure that most of the tubulin is polymerized and therefore is in the pellet fraction.

**Microtubule decoration for negative staining electron microscopy**

Microtubules were polymerized as described above. After polymerization, MTs were diluted in GTB + 20 µM paclitaxel to a final concentration of 0.25 mg/ml. MTs were then spun over 100 µl of CB at 165,000 x g for 10 min at 25°C. Supernatant was removed, and the walls of the tube were cleaned with warm GTB buffer to remove any soluble tubulin. After removing the CB, the pellet was carefully washed with warm GTB + 20 µM paclitaxel and resuspended in the same volume of warm GTB + 20 µM paclitaxel as the initial reaction. 5 µl of MTs were then absorbed on plasma cleaned carbon-coated copper grids (400 mesh, Electron Microscopy Sciences; CF400-CU) for 30 sec and washed twice with GTB + 20 µM paclitaxel. GFP-tagged full-length CGNL1 (5 ml, 0.5 mg/ml) were then added on the grid and incubated for 1 min, washed twice with GTB + 20 µM paclitaxel and stained for 1 min with 1% uranyl acetate, blotted and allowed to air dry. Samples were imaged with a Talos L120C microscope (120 KeV, single tilt holder, Thermo Fisher Scientific).

**Immunoblotting**

For immunoblotting (IB), SDS-PAGE gels were loaded with appropriate samples in SB and migration was carried out at 4°C. Proteins were then transferred onto a nitrocellulose membrane (0.45 µm) for 80 min at 100 V at 4°C. Blots were blocked for 1 h in Tris-buffered saline/Tween-20 (TBST) 0.1%/low-fat milk 5% for 1 h before overnight incubation at 4°C with primary antibody (diluted in TBST 0.1%/low-fat milk 3%). After three TBST washes (10 min each), secondary HRP-labeled antibody diluted in TBST 0.1%/low-fat milk 3% were then incubated for 1 h, at RT and washed three times afterwards with TBST (10 min each). Chemiluminescence (ECL) was detected using Amersham ImageQuant 800 (Cytiva). Numbers on the left of immunoblots correspond to sizes in kilodaltons (kDa) of pre-stained markers.

**Quantifications and statistical analysis**

For the quantification of the ratio of pelleted to total CGNL1 and CGN fragments by IB, the chemiluminescence signal intensity of GST was determined in the pellet and the supernatant fractions, with or without microtubules, using Fiji/ImageJ. Quantification was performed on data from at least three separate experiments.

Data processing and analysis were performed using GraphPad Prism. All experiments were carried out at least three times. Statistical significance of quantitative data was determined by two-way ANOVA with post hoc Sidak's test (for multiple comparisons), (ns = not significant, p > 0.5, significant, ∗p ≤ 0.05, ∗∗p ≤ 0.01, ∗∗∗p ≤ 0.001, and ∗∗∗∗p ≤ 0.0001). All graphs are represented as mean ± SD.

## Reagents

**Table:** 

**REAGENT or RESOURCE**	**SOURCE**	**IDENTIFIER**
Antibodies		
Rabbit polyclonal anti-GST tag	Thermo Fisher Scientific	71-7500 RRID: AB_2533994
Mouse monoclonal anti-β-tubulin	Thermo Fisher Scientific	Cat# 32-2600 RRID: AB_2533072
Anti-Mouse IgG (H+L), HRP Conjugate	Agilent Technologies	Cat# P044701-2 RRID: AB_2617137
Anti-Rabbit IgG (H+L), HRP Conjugate	Agilent Technologies	Cat# P044801-2 RRID: AB_2617138
Plasmids		
**CGNL1**		
pGEX4T1-h CGNL1 (1-250)	Citi laboratory, (Guillemot et al. 2008)	S1023
pGEX4T1-h CGNL1 (250-420)	Citi laboratory, (Guillemot et al. 2008)	S1262
pGEX4T1-h CGNL1 (421-603)	Citi laboratory, (Guillemot et al. 2008)	S1020
pGEX4T1-h CGNL1 (Δ365-377)	Citi laboratory, This paper	S3006
pACEBac1-2xStrep-10xHis-TEV-EGFP-h CGNL1 (FL)	Citi laboratory, This paper	S2962
**CGN**		
pGEX4T1-h CGN (1-406)	Citi Laboratory, (Mangan et al. 2016)	S562
**Control**		
pGEX4T1	Citi laboratory, (Guillemot et al. 2008)	S0050
Critical Commercial Assays and Consumables		
Q5 High fidelity Polymerase	NEB	Cat# M0491L
Porcine tubulin	Cytoskeleton Inc.	Cat# T240
GTP	Cytoskeleton Inc.	Cat# BST06
Paclitaxel	Focus Biomolecules	Cat# FBM-10-2095
Carbon coated copper grids, 400 mesh	Electron Microscopy Sciences	Cat# CF400-CU
Pierce Protease inhibitor cocktail	Thermo Fisher Scientific	A32963
Pierce glutathione magnetic agarose beads	Thermo Fisher Scientific	Cat# 78602
Experimental Models: Organisms/Strains		
BL21 Competent cells	NEB	Cat# C2530H
Software and Algorithms		
Image J/FIJI	NIH	imagej.nih.gov/ij/
Adobe Photoshop	Adobe	RRID: SCR _014199
Adobe Illustrator	Adobe	RRID: SCR _010279
Prism 8	GraphPad	RRID: SCR _002798
SnapGene	N/A	RRID: SCR _015052

## Extended Data

Description: SDS-PAGE analysis of whole lysate from Sf9 insect cells expressing GFP-CGNL1 (lane 1), purified GFP-CGNL1 (lane 2) and molecular weight markers (lane 3). Sizes in kDa are indicated on the right.. Resource Type: Image. DOI: 10.22002/5qywn-r7n90
